# Brain Endothelial P-Glycoprotein Level Is Reduced in Parkinson’s Disease via a Vitamin D Receptor-Dependent Pathway

**DOI:** 10.3390/ijms21228538

**Published:** 2020-11-12

**Authors:** Hyojung Kim, Jeong-Yong Shin, Yun-Song Lee, Seung Pil Yun, Han-Joo Maeng, Yunjong Lee

**Affiliations:** 1 Department of Pharmacology, Sungkyunkwan University School of Medicine, Suwon 16419, Korea; hjung93@skku.edu (H.K.); wjddydehtk@skku.edu (J.-Y.S.); yslee@skku.edu (Y.-S.L.); 2 Department of Pharmacology and Convergence Medical Science, College of Medicine, Gyeongsang National University, Jinju 52727, Korea; spyun@gnu.ac.kr; 3 College of Pharmacy, Gachon University, Incheon 21936, Korea

**Keywords:** vitamin D receptor, Parkinson’s disease, P-glycoprotein, 6-hydroxydopamine, brain endothelium, α-synuclein aggregation

## Abstract

The progressive neurodegeneration in Parkinson’s disease (PD) is accompanied by neuroinflammation and endothelial vascular impairment. Although the vitamin D receptor (VDR) is expressed in both dopamine neurons and brain endothelial cells, its role in the regulation of endothelial biology has not been explored in the context of PD. In a 6-hydroxydopamine (6-OHDA)-induced PD mouse model, we observed reduced transcription of the VDR and its downstream target genes, *CYP24* and *MDR1a*. The 6-OHDA-induced transcriptional repression of these genes were recovered after the VDR ligand—1α,25-dihydroxyvitamin D_3_ (1,25(OH)_2_D_3_) treatment. Similarly, reduced vascular protein expression of P-glycoprotein (P-gp), encoded by *MDR1a*, after 6-OHDA administration was reversed by 1,25(OH)_2_D_3_. Moreover, marked reduction of endothelial P-gp expression with concomitant α-synuclein aggregation was found in a combinatorial *AAV-αSyn*/αSyn preformed fibril (PFF) injection mouse model and postmortem PD brains. Supporting the direct effect of α-synuclein aggregation on endothelial biology, PFF treatment of human umbilical vein endothelial cells (HUVECs) was sufficient to induce α-synuclein aggregation and repress transcription of the VDR. PFF-induced P-gp downregulation and impaired functional activity in HUVECs completely recovered after 1,25(OH)_2_D_3_ treatment. Taken together, our results suggest that a dysfunctional VDR-P-gp pathway could be a potential target for the maintenance of vascular homeostasis in PD pathological conditions.

## 1. Introduction

Parkinson’s disease (PD) is mainly characterized by the rather selective and progressive demise of nigral dopaminergic neurons, which leads to the cardinal motor impairment in patients [1,2]. Pathologically, nondopaminergic neurons and various brain regions are also influenced by the progression of the disease and propagation of Lewy body pathology [3,4]. As the main component of Lewy bodies or Lewy neurites, which are the diagnostic hallmark of PD and related α-synucleinopathies, pathological α-synuclein aggregates can propagate into many brain regions and interact with diverse cell types [5,6,7]. α-synuclein preformed fibrils (PFFs) have been shown to interact with Lag3A and can be taken up by neurons [7], thereby explaining the neuron-to-neuron transmission of α-synuclein pathologies. In addition to its effect on neurons, α-synuclein aggregates also bind to the Toll-like receptor (TLR) expressed on microglia [5]. This binding of α-synuclein aggregates with the TLR stimulates microglial activation and inflammatory mediator release, which can modulate astrocyte conversion and neuroinflammation [8]. 

Studies have also found pathological alteration of endothelial cells lining the cerebral blood vessels in PD [9,10]. Brain endothelial degeneration with surrounding α-synuclein pathologies has been seen in postmortem brain tissues in PD [10]. Moreover, in a previous study, α-synuclein PFF treatment of cultured cerebral endothelial cells led to altered expression of tight junction proteins [11], suggesting a pathological role of α-synuclein pathology in endothelial function. Blood vessel endothelial cells also express membrane transporters including P-glycoprotein (P-gp), which is involved in the regulation of the exchange of metabolites, drugs, and peptides, such as amyloid beta, between the brain and peripheral bloodstream through the blood–brain barrier (BBB) [12,13]. P-gp is encoded by multidrug resistance protein 1 (*MDR1*) genes. Although P-gp downregulation and deficits have been reported in postmortem PD brain samples [14,15], its underlying mechanisms are largely unexplored.

The vitamin D receptor (VDR), a nuclear receptor, responds to the active vitamin D metabolite—1α,25-dihydroxyvitamin D_3_ (1,25(OH)_2_D_3_; also known as calcitriol)—and vitamin D analogs and plays several important biological roles in the body. Expression of the VDR in dopamine neurons and brain endothelial cells indicates its extensive role in the regulation of brain environments by affecting gene expression in diverse cell types. Vitamin D deficiency has been associated with an increased risk of developing PD [16]. Treatment with VDR ligand drugs has been shown to prevent PD-related phenotypes in 6-OHDA-induced rat models of PD [17]. The ligand-bound VDR can regulate pathways involved in the expression of antioxidant enzymes, such as glutathione synthesis [18], and the expression of neurotrophic factors [19], which could explain the neuroprotection provided by VDR activation against 6-OHDA-induced neurotoxicity. In addition, the VDR regulates the expression of drug transporters and metabolic enzymes [20,21]. Although studies regarding the regulation of drug transporters and/or expression have focused on the intestine, liver, and kidney [21,22,23], limited studies have been conducted to evaluate the role of the VDR in regulation of drug transporters in the BBB. The expression of the membrane efflux transporter P-gp is increased by VDR activation in endothelial cell models and rodent brains [20,24]. Functional vitamin D response elements in the human *MDR1* gene encoding P-gp have also been identified [25]. Although the link between VDR activation and P-gp expression in endothelial cells is well established, it is unclear whether potential dysregulation of the VDR-P-gp pathway under PD pathological conditions (e.g., oxidative stress and α-synuclein aggregation) can be reversed by VDR ligand treatment.

In this study, we identified VDR repression in 6-OHDA and α-synucleinopathy PD mouse models. Consistent with VDR downregulation, the VDR target gene endothelial P-gp expression was substantially reduced in response to 6-OHDA stress or α-synuclein aggregate pathologies in vivo. VDR activation after 1,25(OH)_2_D_3_ treatment prevented 6-OHDA-induced dopamine cell loss and neuroinflammation with concomitant recovery of P-gp expression in CD31-labeled brain endothelial cells. Mechanistically, α-synuclein PFF treatment of human umbilical vein endothelial cells (HUVECs) and its uptake caused impairment of the VDR pathway by downregulating VDR transcription. PFF-induced P-gp downregulation in HUVECs is VDR dependent since 1,25(OH)_2_D_3_ treatment completely prevented this change. Since we observed α-synuclein aggregation and P-gp downregulation in PD postmortem brains, activating the VDR pathway might be a potential therapeutic strategy to restore BBB dysfunction in PD.

## 2. Results

### 2.1. VDR Stimulation by 1,25(OH)_2_D_3_ Is Neuroprotective in 6-OHDA PD Mice

**Figure 1:**
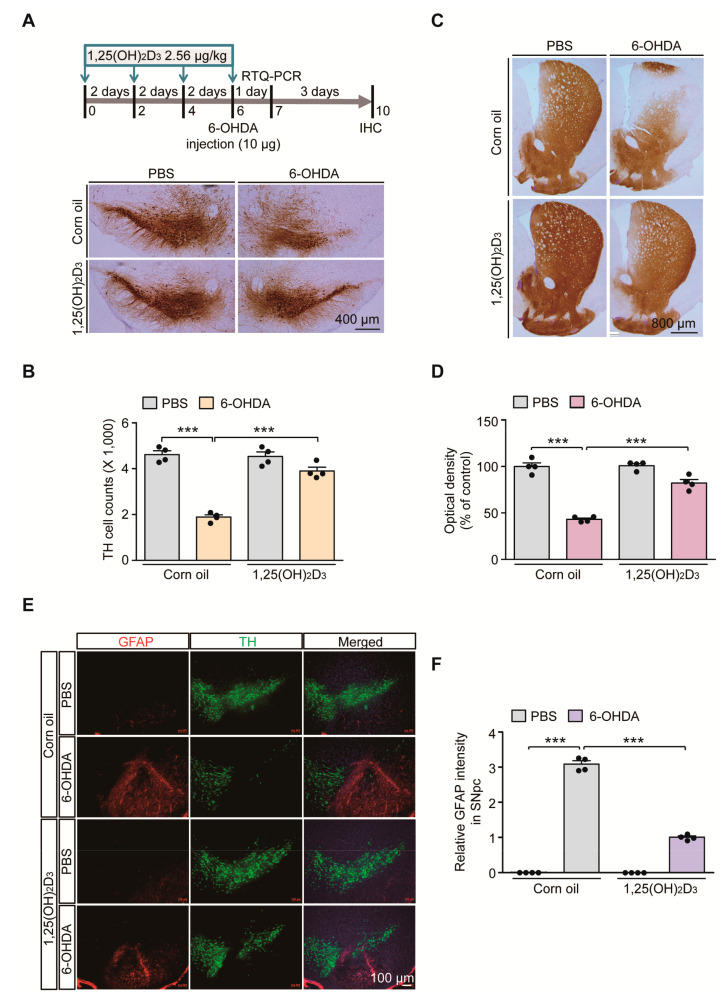
1,25(OH)_2_D_3_ treatment prevents dopaminergic loss and neuroinflammation in a 6-hydroxydopamine (6-OHDA) Parkinson’s disease (PD) mouse model. (**A**) Experimental schedule of 6-OHDA unilateral striatal injection to model PD in mice and 1,25(OH)_2_D_3_ intraperitoneal administration (upper panel). 1,25(OH)_2_D_3_ was administered once every two days (total four times) followed by stereotaxic 6-OHDA injection into the striatum of the right hemisphere. Total RNA from the ventral midbrain was prepared for RTQ-PCR application one day after the 6-OHDA striatal injection. Mouse brains were PFA fixed for immunolabeling experiments four days after the 6-OHDA striatal injection. (**B**) Representative anti-TH immunohistochemistry of ventral midbrain sections from mice in each indicated experimental group. Scale bar = 400 μm. (B) Stereological assessment of TH-positive cell counts in the substantia nigra pars compacta from each mouse group (*n* = 4 mice per group). (**C**) Representative anti-TH immunohistochemistry of the striatum from the indicated mouse group. Scale bar = 800 μm. (**D**) Quantification of optical densities of anti-TH stained dopaminergic nerve terminal in the striatum from each mouse group (*n* = 4 mice per group). (**E**) Representative immunofluorescence of GFAP and TH in the ventral midbrain sections from each experimental mouse group. Scale bar = 100 μm. (**F**) Quantification of GFAP immunofluorescence signal intensities in the SNpc regions from each mouse group (*n* = 4 mice per group). Data are expressed as mean ± standard error of the mean (SEM). *** *p* < 0.001, ANOVA, followed by Tukey’s post hoc analysis.

Vitamin D signaling pathways are involved in diverse biological processes and its dysfunction is implicated in the pathogenesis of PD [16]. To determine the potential dysregulation of VDR signaling pathways in PD-like conditions, we employed the 6-OHDA-induced PD mouse model. To model oxidative stress-induced dopaminergic neuronal loss, 6-OHDA was stereotaxically introduced into the striatum of 3-month-old C57/BL6J mouse brains (Figure 1A). The biologically active form of vitamin D, 1,25(OH)_2_D_3_, was intraperitoneally administered every other day to pharmacologically stimulate VDR signaling pathways in mice (Figure 1A). Consistent with previous findings in rat 6-OHDA-induced PD models [17], robust loss of TH-stained dopamine neurons in the SNpc after striatal injection of 6-OHDA was largely prevented by 1,25(OH)_2_D_3_ pretreatment, determined using the unbiased stereological assessment (Figure 1A,B). Correlating with the dopamine cell count in the SN region, there was marked reduction of TH-positive dopaminergic nerve terminals in the striatum of 6-OHDA-injected mice (Figure 1C,D). A pretreatment with 1,25(OH)_2_D_3_ prevented this 6-OHDA-induced loss of striatal dopaminergic nerve fibers (Figure 1C,D). 

With this nigrostriatal dopaminergic loss, the robust manifestation of neuroinflammation was noted in 6-OHDA PD mouse ventral midbrains, evidenced by the enhanced immunofluorescence signals of GFAP-stained astrogliosis (Figure 1E,F). An increased GFAP signal was obvious in the degenerating environments of SN regions with fewer TH-stained dopamine neurons (Figure 1E). GFAP signal enhancement in the 6-OHDA model was largely blocked by 1,25(OH)_2_D_3_ treatment (Figure 1E,F). Taken together, these results indicate that oxidative stress-induced dopamine cell loss and neuroinflammation can be substantially deterred by VDR activation in mouse brains.

### 2.2. 1,25(OH)_2_D_3_ Treatment Restores 6-OHDA-Induced Impairment of VDR-Endothelial P-gp Signaling Pathway In Vivo

**Figure 2:**
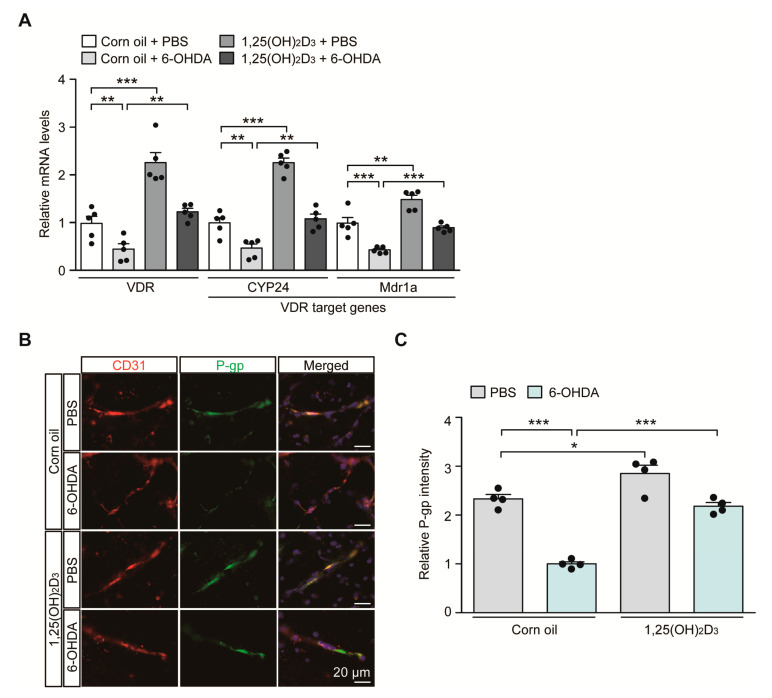
Transcriptional VDR repression and endothelial P-glycoprotein (P-gp) protein downregulation in 6-OHDA PD mice are reversed by 1,25(OH)2D3 treatment. (**A**) Quantification of relative mRNA expression of VDR and its target genes *CYP24* and *MDR1a* in the ventral midbrain tissues from each experimental mouse group determined using qRT-PCR and normalized to internal loading control GAPDH (*n* = 5 mice per group). (**B**) Representative immunofluorescence of P-gp and CD31 in the ventral midbrain sections from each experimental mouse group. CD31 serves as a marker for endothelial cells. Scale bar = 20 μm. (**C**) Quantification of P-gp immunofluorescence signal intensities in CD31-positive endothelial cells from the SNpc regions from each mouse group (*n* = 4 mice per group). Data are expressed as mean ± SEM. * *p* < 0.05, ** *p* < 0.01, and *** *p* < 0.001, ANOVA test, followed by Tukey’s post hoc analysis.

In the 6-OHDA PD mouse model with the induction of characteristic PD pathology, we sought to monitor the transcriptional regulation of the VDR itself and its target genes *CYP24* and *MDR1a*, which play important roles in brain vascular endothelial cell function. Consistent with the results of previous reports showing downregulation of VDR protein expression in 6-OHDA-intoxicated rodents [17], we saw an approximate 50% reduction in *VDR* mRNA expression in the ventral midbrain of mice after the 6-OHDA striatal injection, monitored using quantitative RT (qRT)-PCR (Figure 2A). This VDR repression is correlated with downregulation of the well-established VDR target genes *CYP24* and *MDR1a* in 6-OHDA injected mouse brains (Figure 2A). VDR stimulation after 1,25(OH)_2_D_3_ administration resulted in a more than two-fold increase in mRNA levels of *VDR* and *CYP24* and an approximately 50% increase in MDR1a mRNA levels in PBS-injected control mice (Figure 2A). 1,25(OH)_2_D_3_ pretreatment prevented 6-OHDA-induced repression of *VDR*, *CYP24*, and *MDR1a* transcription and maintained transcription of these genes at levels comparable to those in the control group (Figure 2A). 

Since *MDR1a* encodes the membrane transporter P-gp, which is mainly expressed in vascular endothelial cells, we determined P-gp expression in the brains of 6-OHDA PD mouse models with or without 1,25(OH)_2_D_3_ treatment at the cellular resolution using immunofluorescence. Consistent with the qRT-PCR results, there was an approximately 50% reduction in P-gp expression in CD31-labeled vascular endothelial cells in the ventral midbrains of dopaminergic neurodegeneration in response to striatal 6-OHDA injection (Figure 2B,C). We further observed concomitant and marked downregulation of endothelial expression of VDR, the upstream regulator of P-gp in 6-OHDA-induced PD mice (Figure S1A,B). Treatment with 1,25(OH)_2_D_3_ abolished this endothelial P-gp and VDR downregulation in PD pathological environments elicited by 6-OHDA injection (Figure 2B,C, and Figure S1A,B). 1,25(OH)_2_D_3_ has the ability to enhance endothelial P-gp and VDR protein expression even in PBS-injected control mice (Figure 2B,C, and Figure S1A,B).

### 2.3. Clinical Relevance of Endothelial P-gp Downregulation with α-Synuclein Pathology in PD

**Figure 3:**
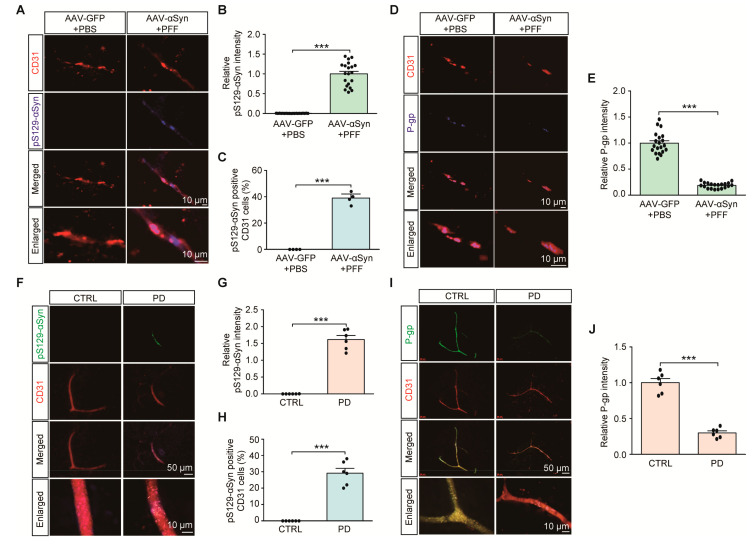
Endothelial α-synuclein aggregation and reduction of P-gp expression in PD. (**A**) Representative immunofluorescence of S129 phosphorylated α-synuclein (pS129-α-Syn) and CD31 in the ventral midbrain sections from mice with intranigral coinjections of *AAV-αSyn* (human) and αSyn preformed fibril (PFF). *AAV-GFP* (*AAV-Control*) and PBS were used as control injections. Scale bar = 10 μm. (**B**) Quantification of pS129-α-Syn immunofluorescence signal intensities in CD31-positive endothelial cells from the SNpc from each mouse group (*n* = 20 sections from 4 mice per group). (**C**) Percentage of CD31-positive cells with pS129-α-Syn pathologies in each mouse group (*n* = 4 mice per group). (**D**) Representative immunofluorescence of P-gp and CD31 in the ventral midbrain sections from each experimental mouse group. Scale bar = 10 μm. (**E**) Quantification of P-gp immunofluorescence signal intensities in CD31-positive endothelial cells from the SNpc from each mouse group (*n* = 20 sections from 4 mice per group). (**F**) Representative immunofluorescence of S129 phosphorylated α-synuclein (pS129-α-Syn) and CD31 in the cortex sections from postmortem human brains of patients with PD and age-matched healthy controls. Scale bar = 50 or 10 μm. (**G**) Quantification of pS129-α-Syn immunofluorescence signal intensities in CD31-positive endothelial cells from postmortem cortex sections from postmortem human brains of patients with PD and age-matched healthy controls *n* = 6 Con, *n* = 6 PD). (**H**) Percentage of CD31-positive cells with pS129-α-Syn pathologies in each group (*n* = 6 Con, *n* = 6 PD). (**I**) Representative immunofluorescence of P-gp and CD31 in the postmortem human cortical sections from each group. Scale bar = 50 or 10 μm. (**J**) Quantification of P-gp immunofluorescence signal intensities in CD31-positive endothelial cells from each group (*n* = 6 Con, *n* = 6 PD). Data are expressed as mean ± SEM. *** *p* < 0.001, unpaired two-tailed Student’s *t* test.

We next examined the potential dysregulation of brain vascular endothelial P-gp expression in PD-associated pathological conditions, such as α-synuclein aggregation. With some modification, we employed a sporadic α-synucleinopathy PD mouse model with combinatorial nigral injections of PFF with AAV expressing human α-synuclein (*AAV-αSyn*) (Figure S2A). In this combinatorial PD mouse model of α-synucleinopathy, a robust expression of neuronal α-synuclein aggregation was achieved, shown by the enhanced immunofluorescence of phosphorylated α-synuclein (pS129-αSyn), a common indicator of α-synuclein aggregation (data not shown). In addition to this neuronal α-synuclein aggregation pathology, the combinatorial PD mouse model exhibited the presence of pS129-αSyn positive aggregates in CD31-stained endothelial cells (Figure 3A,B). Approximately 40% of all CD31 endothelial cells expressed pS129-αSyn pathologies (Figure 3C). Under these pathological circumstances of α-synuclein aggregation, there was an approximately 90% reduction in VDR, and an 80% reduction in P-gp endothelial expression in the ventral midbrains of combinatorial PD mouse models compared to that in the ventral midbrains of control mice (Figure S2B,C, and Figure 3D,E). 

We observed the presence of pS129-αSyn expression in brain vascular endothelial cells from PD postmortem brain tissues (Figure 3F,G, Table 1). Approximately 30% endothelial cells exhibited α-synuclein aggregation pathologies (Figure 3H). Moreover, consistent with the results of a previous report [15], there was marked reduction in P-gp endothelial expression in brain samples of postmortem PD patients compared to that in brain samples of age-matched healthy controls (Figure 3I,J). These results indicate potential brain vascular dysfunction with characteristic α-synuclein aggregation and transporter P-gp deficits in PD.

### 2.4. PFF-Induced Repression of the VDR-P-gp Pathway in HUVECs Is Restored by 1,25(OH)_2_D_3_

**Figure 4:**
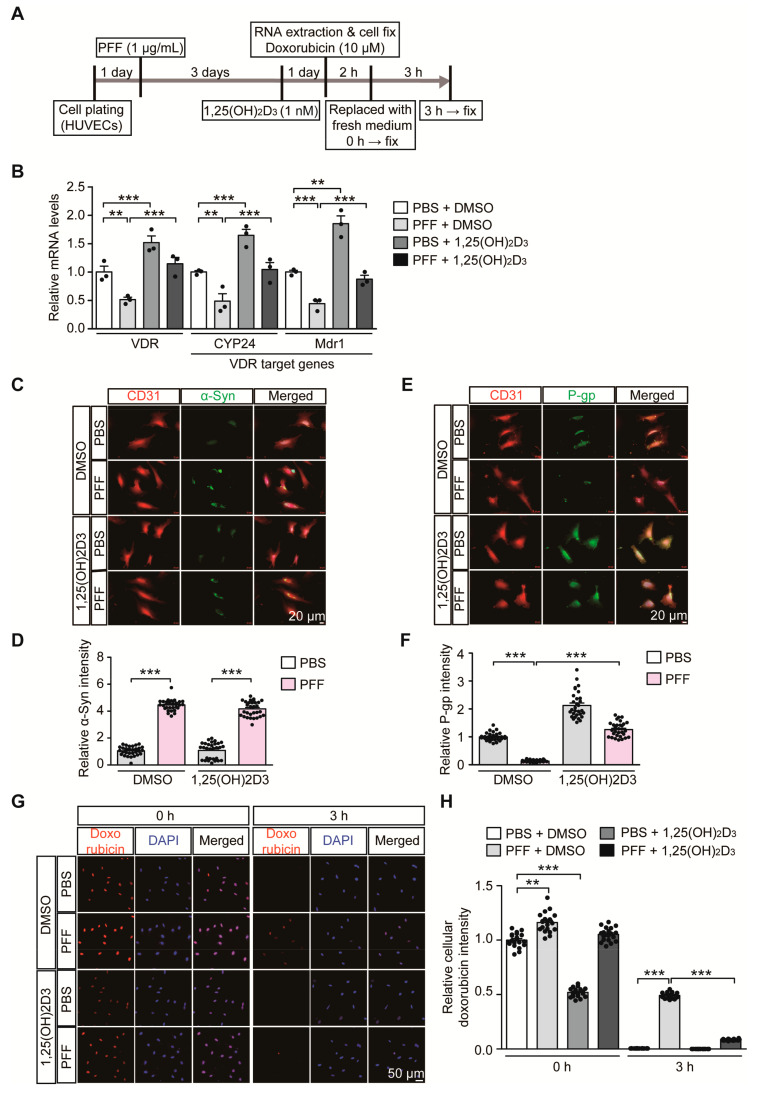
1,25(OH)_2_D_3_ activation of VDR reverses VDR transcriptional downregulation and reduced P-gp expression in HUVECs following PFF treatment. (**A**) Experimental schedule of RTQ-PCR and immunofluorescence analysis for PFF-induced α-synucleinopathy HUVEC model with or without 1,25(OH)_2_D_3_ post-treatment. Experimental schedule of the subsequent transporter functional assay using doxorubicin as P-gp substrate was also indicated. (**B**) Quantification of relative mRNA expression of VDR and its target genes, *CYP24* and *MDR1*, in HUVECs treated with PFF or PBS as a control determined using qRT-PCR and normalized to internal loading control GAPDH (*n* = 3 per group). (**C**) Representative immunofluorescence of α-synuclein and CD31 in the HUVECs treated with PFF (1 µg/mL, 4 days) or PBS in the presence or absence of 1,25(OH)_2_D_3_ (1 nM, 24 h). Scale bar = 20 μm. (**D**) Quantification of α-Syn immunofluorescence signal intensities in CD31-stained HUVECs from each experimental group (*n* = 30 cells per group). (**E**) Representative immunofluorescence of P-gp and CD31 in HUVECs given the indicated treatments. Scale bar = 20 μm. (**F**) Quantification of P-gp immunofluorescence signal intensities in CD31-stained HUVECs from each experimental group (*n* = 30 cells per group). (**G**) Representative fluorescence images of intracellular doxorubicin at the indicated time points (0, and 3 h) in HUVECs following preincubation with 10 uM doxorubicin (2 h). DAPI was used to counterstain the nucleus. (**H**) Quantification of relative intracellular doxorubicin autofluorescence in HUVECs at the indicated time points following 10 uM doxorubicin preincubation (2 h) (*n* = 20 cells from 3 experiments per group). Data are expressed as mean ± SEM. ** *p* < 0.01, and *** *p* < 0.001, ANOVA test, followed by Tukey’s post hoc analysis.

Based on our observation of correlative alteration of α-synuclein aggregation and P-gp expression, we sought to determine the pathological role of α-synuclein aggregation on the VDR-P-gp pathway in endothelial cells. HUVECs were used to model a PD-relevant pathological environment with the challenge of extracellular α-synuclein PFF treatment (Figure 4A). Similar to 6-OHDA stress in mice, PFF treatment in HUVECs caused downregulation of *VDR* transcription with concomitant reduction of its target genes, *CYP24* and *MDR1* (Figure 4B). These alterations in *CYP24* and *MDR1* after PFF treatment were reversed to the basal levels seen in controls after VDR activation by 1,25(OH)_2_D_3_ treatment (Figure 4B). 1,25(OH)_2_D_3_ treatment in the control group with no PFF treatment resulted in an approximately 50% increase in *VDR* transcription and subsequent *CYP24* and *MDR1* upregulation, indicating that the VDR-CYP24/MDR1 signaling pathway is active in HUVECs.

PFF treatment resulted in increased intracellular α-synuclein immunofluorescence signals in HUVECs (Figure 4C,D, and Figure S3A), indicating the potential uptake of PFF into HUVECs. PFF treatment in HUVECs and accumulation of intracellular α-synuclein led to marked downregulation of P-gp expression (Figure 4E,F, and Figure S3B), which was rescued by 1,25(OH)_2_D_3_ treatment. Although 1,25(OH)_2_D_3_ had no effect on the uptake of PFF into HUVECs (Figure 4C,D, and Figure S3A), it prevented PFF-induced P-gp downregulation in HUVECs (Figure 4E,F, and Figure S3B). In each experimental condition with the relatively short duration of PFF or 1,25(OH)_2_D_3_ treatment, there were no observable morphological signs of obvious cytotoxicity 

To examine the membrane transporter functions in HUVECs, we monitored time course uptake and clearance of doxorubicin in HUVECs. Doxorubicin serves as a substrate for P-gp and can emit autofluorescence, thus facilitating the measurement of its cellular accumulation [26]. In our experimental setting of low dose and brief doxorubicin preincubation (10 μM, 2 h), there was no demonstrable cytotoxicity in HUVECs as assessed by Cell Counting Kit-8 cell viability assay (Figure S4A). Under normal conditions, doxorubicin preincubation (10 μM, 2 h) resulted in substantial intracellular accumulation of doxorubicin in HUVECs (Figure S4B,C). The intracellular doxorubicin remained high up to 1 h, but its levels gradually decreased with no detectable doxorubicin fluorescence signal at 4 h time point (Figure S4B,C). This efflux of doxorubicin in HUVECs seems to be mainly mediated by P-gp since P-gp inhibition by verapamil treatment [27,28] largely blocked doxorubicin elimination from HUVECs (Figure S4B,C). Consistent with our observation that PFF treatment caused P-gp downregulation in HUVECs (Figure 4E,F), there was delayed removal of doxorubicin in PFF-treated HUVECs as compared to robust doxorubicin clearance in control HUVECs (Figure 4G,H), indicating diminished P-gp efflux function in HUVECs by PFF treatment. The impaired P-gp efflux function by PFF stress in HUVECs was largely recovered by 1,25(OH)_2_D_3_ treatment (Figure 4G,H). Interestingly, control HUVECs with 1,25(OH)_2_D_3_ treatment showed lower steady state doxorubicin accumulation at 0 h time point (Figure 4G,H). This is consistent with enhanced P-gp protein expression by 1,25(OH)_2_D_3_ treatment under the basal condition (Figure 4E,F). However, despite the marked downregulation of P-gp expression by PFF treatment, we only observed modest increase of doxorubicin accumulation at 0 h time point (Figure 4G,H), suggesting potential involvement of another transporter expression in response to PFF treatment. Taken together, these results suggest the direct pathological effect of α-synuclein aggregates on endothelial VDR signaling pathways and transporter function.

## 3. Discussion

This is the first study to report on the molecular mechanism by which PD-relevant oxidative stress and α-synuclein aggregation influences brain vascular biology. 6-OHDA or PFF led to transcriptional repression of the *VDR* and its target genes, especially P-gp in mouse brains and in HUVECs. It is unclear how these two distinct stresses caused repression of *VDR* transcription. However, both oxidative stress and α-synuclein aggregation are implicated in the pathogenesis of PD and thought to have a pathological reciprocal interaction. Furthermore, 6-OHDA-induced oxidative stress is known to prompt α-synuclein aggregation [29]. Conversely, α-synuclein aggregation can affect many biological processes through sequestering functionally important proteins [30]. In particular, the pathological interaction of α-synuclein with the mitochondria [31] might have resulted in an increase in oxidative stress. It is likely that there are common regulators of VDR that are dysregulated by oxidative stress and α-synuclein aggregation. Since the protein kinase A (PKA) pathway is involved in the transcription of VDR through the sp1 site of VDR promoter [32], it would be informative to examine the potential dysregulation of PKA pathways in response to 6-OHDA or PFF in HUVECs. Interestingly, we showed that 1,25(OH)_2_D_3_ treatment stimulated transcription of the *VDR* gene in the mouse brains and HUVECs. It seems that *VDR* promoter activation is controlled by interplay of multiple factors depending on different cell types because there are conflicting results on VDR transcriptional activation by 1,25(OH)_2_D_3_ treatment [12,17,33]. Overall, although there have been several reports regarding VDR or P-gp regulation in PD [14,15,16], our result links the endothelial VDR-P-gp regulation pathway to PD pathological conditions. Although our study has mainly focused on regulation of endothelial VDR-P-gp mRNA or protein expression, detailed assessment for subcellular distribution of VDR and P-gp under PD-relevant stresses might provide further mechanistic insights into endothelial functional impairment in PD. 

In our PD mouse models and human brain samples, the mRNA/protein expression of P-gp in the BBB was significantly lower than that in the vehicle-treated group, accompanying impaired VDR activation. Collectively, these data suggest that in PD, P-gp can be markedly downregulated, likely resulting in reduced P-gp efflux at the BBB. Considering that substrates for P-gp include toxic endogenous substrates, xenobiotics, and many clinically important drugs, such as anticancer, antiviral, and central nervous system-related drugs, impaired P-gp efflux function at the BBB in PD is likely to elevate the levels of toxic endogenous substrates and xenobiotics. In addition, it can be reasonably inferred that possible changes in the brain concentration of P-gp substrate drugs occur in PD patients. In a previous case report, higher brain penetration of [^11^C]-verapamil in vivo was noted in PD patients on positron emission tomography [15], suggesting impaired P-gp function in PD patients. Further pharmacokinetic studies regarding impaired P-gp function due to decreased P-gp expression in the brain are required. In this regard, it would be instructive to evaluate therapeutic potential of 1,25(OH)_2_D_3_ in sporadic PD mouse model of α-synucleinopathy with combinatorial brain injections of PFF and *AAV-αSyn* [34]. Since this chronic PD mouse model exhibits both progressive dopaminergic neuron loss and vascular pathologies with reduced P-gp expression, preclinical studies with 1,25(OH)_2_D_3_ post-treatment would validate translational value of the VDR signaling pathway in treating diverse aspects of PD pathogenesis. Moreover, P-gp has been implicated in the efficient efflux of disease protein aggregates, such as amyloid beta, from the brain to the blood circulation in mouse models of Alzheimer’s disease [12]. Similarly, PD-associated α-synuclein has been shown to be transported from the brain to the blood circulation [35]. Further investigation is necessary to determine whether VDR regulation of endothelial transporters, including P-gp, plays a role in the clearance of intracerebral α-synuclein load in PD. Since 1,25(OH)_2_D_3_ treatment significantly recovered P-gp expression at the BBB in the 6-OHDA PD mouse model and in vitro HUVEC model treated with PFF, VDR activation may be considered a therapeutic target to recover P-gp expression and, thus, vascular function in PD. The in vitro doxorubicin efflux assay showed that basal clearance of doxorubicin in HUVECs is mainly mediated by P-gp activity. However, our doxorubicin-mediated assessment of P-gp transporter function in PFF-induced PD HUVECs model needs to be supplemented with additional control experiments including efflux assays using other non-cytotoxic P-gp substrates, pharmacological inhibition with other P-gp inhibitors, and genetic ablation of P-gp to address potential nonspecific effects by P-gp inhibitors. 

Although our current study focused on endothelial P-gp regulation by VDR in PD environment, VDR deficits in PD might influence diverse physiological functions of endothelial cells in the BBB. Disruption of the BBB in the postmortem examination of brains of patients with PD has been noted with the penetration of autoantibodies and T lymphocyte into the lesioned brain area [36]. Further, PFF treatment of the cerebral endothelial cell line can disrupt endothelial functions with a reduction in endothelial tight junction proteins, occludin and ZO-1 [11]. They showed the clinical relevance of downregulation of occludin and ZO-1 in postmortem PD brain samples relative to that in control samples [11]. Since VDR is also involved in the regulation of the expression of several tight junctions [37], it would be instructive to further investigate the potential dysregulation of these genes in endothelial cells in a PD environment and whether these pathological alterations could be normalized by VDR activation. Maintenance of the endothelial tight junction and BBB integrity due to VDR activity is important in preventing pathological brain immune responses in PD. 

## 4. Materials and Methods

### 4.1. Chemicals and Antibodies

1,25(OH)_2_D_3_ and 6-OHDA were purchased from Sigma-Aldrich (1,25(OH)_2_D_3_; 17936, 6-OHDA; H4381, Sigma-Aldrich, St. Louis, MO, USA). The following primary antibodies were used: rabbit antibody to tyrosine hydroxylase (TH) (NB300-109, 1:2000, Novus Biologicals, Littleton, CO, USA), rabbit antibody to GFAP (ab7260, 1:1000, Abcam, Cambridge, UK), rabbit antibody to CD31 (ab28364, 1:1000, Abcam, Cambridge, UK), mouse antibody to CD31 (#550274, 1:1000, BD biosciences, Franklin lakes, NJ, USA), rabbit antibody to VDR (ab3508, 1:1000, Abcam, Cambridge, UK), mouse antibody to P-gp (sc-55510, 1:1000, Santa Cruz, Dallas, TX, USA), mouse antibody to pS129-α-synuclein (#825701, Biolegend, San Diego, CA, USA), and mouse antibody to α-synuclein (BD-610787, BD Biosciences, Franklin lakes, NJ, USA). 

The following secondary antibodies were used: biotin-conjugated goat antibody to rabbit IgG (cat# BA-1000, 1:1000, Vector Laboratories, Burlingame, CA, USA), Alexa fluor 568-conjugated donkey antibody to rabbit IgG (A10042, Invitrogen, Carlsbad, CA, USA), Alexa fluor 488-conjugated donkey antibody to mouse IgG (A21202, Invitrogen, Carlsbad, CA, USA), and Alexa Fluor 405-conjugated goat antibody to mouse IgG (A31553, Invitrogen).

### 4.2. HUVEC Culture and Treatment

HUVECs (ATCC#CRL-1730, ATCC, Manassas, VA, USA) were grown in Kaighn’s modification of Ham’s F-12 medium (F-12K) (ATCC#30-2004, ATCC, Manassas, VA, USA) containing 10% fetal bovine serum (FBS) (*v*/*v*), heparin solution (final concentration, 0.1 mg/mL; H3393, Sigma-Aldrich, St. Louis, MO, USA), endothelial cell growth supplement (ECGS, #354006, BD Biosciences, San Jose, CA, USA), and antibiotics (penicillin–streptomycin 100 U/mL; Sigma-Aldrich, St. Louis, MO, USA). Cells were maintained in a humidified atmosphere of 5% CO_2_/95% at 37 °C. These cells were plated in 6-well plates at a density of 0.5 × 10^6^ cells per well and harvested at 4 days after treatment with PFF (final concentration, 1 µg/mL) or phosphate-buffered saline (PBS). DMSO (#276855, Sigma-Aldrich) or 1,25-(OH)_2_D_3_ (Calcitriol, #17936, Sigma-Aldrich) treatment was provided for 24 h prior to harvest.

### 4.3. Real-Time Quantitative Polymerase Chain Reaction

Total RNA extraction, cDNA synthesis and the following real-time polymerase chain reaction (RT-PCR) analysis of target transcript levels were done as described previously [6]. The primer sequences used RT-PCR were as follows:
mGAPDH:F- 5′ TGGCCTTCCGTGTTCCTAC 3′, R- 5′ GAGTTGCTGTTGAAGTCGCA 3′mVDR: F- 5′ GAGGTGTCTGAAGCCTGGAG 3′, R- 5′ ACCTGCTTTCCTGGGTAGGT 3′mCYP24: F- 5′ CTGCCCCATTGACAAAAGGC 3′, R- 5′ CTCACCGTCGGTCATCAGC 3′mMDR1a: F- 5′ CAGCAGTCAGTGTGCTTACAA 3′, R- 5′ ATGGCTCTTTTATCGGCCTCA 3′hGAPDH:F- 5′ AAACCCATCACCATCTTCCAG 3′, R- 5′ AGGGGCCATCCACAGTCTTCT 3′hVDR:F- 5′ GTGGACATCGGCATGATGAAG 3′, R- 5′ GGTCGTAGGTCTTATGGTGGG 3′hCYP24:F- 5′ CGACTACCGCAAAGAAGGCTA 3′, R- 5′ ACCATTTGTTCAGTTCGCTGT 3′hMDR1:F- 5′ GGGAGCTTAACACCCGACTTA 3′, R- 5′ GCCAAAATCACAAGGGTTAGCTT 3′

### 4.4. Animal Experiments

All animal experiments were approved by the Ethical Committee of Sungkyunkwan University (Approval #, SKKUIACUC2019-06-15-1) and were conducted in accordance with all the applicable international guidelines. Male C57BL/6N mice (aged 3 months) were purchased from Orient (Suwon, Korea). Animals were maintained in a 12-h dark/light cycle in air-controlled rooms and were provided ad libitum access to food and water. 1,25-(OH)_2_D_3_ was administered (i.p., 2.56 µg/kg body weight, once every 2 day) on days 0, 2, 4 and 6. 

### 4.5. Stereotaxic Injection of 6-OHDA or PFF/rAAV-αSyn

For stereotaxic injection of 6-OHDA (10 µg), 3-month-old C57BL/6N mice were anesthetized with alfaxan (60 mg/kg) on day 6. The 6-OHDA injection procedure was performed as described previously [38]. The combinatorial α-synucleinopathy PD model was generated by simultaneously injecting PFF (10 µg; ventral tegmental area: AP, −3.4 mm; ML, −0.5 mm; DV, −4.3 mm) and *rAAV-αSyn* (AAV serotype 1, 1 µL of titer 5 × 1011 GC/mL; substantia nigra pars compacta [SNpc]: AP, −3.4 mm; ML, −1.3 mm; DV, −4.3 mm) unilaterally. Similar surgical procedures to those used in the 6-OHDA PD model were followed [34]. *AAV-GFP* was also used in the control experimental group. PFF was prepared from pure recombinant human α-synuclein (Proteos, Inc, Kalamazoo, MI, USA) according to a previous report [39]. Before use, sonicated PFF was evaluated using western blotting and a functional assay in a cortical neuron culture (pS129-αSyn induction and neurotoxicity). 

### 4.6. TH Stereological Cell Counting

After intracardial fixation of mice, we followed the previously reported procedure [38] for brain coronal sectioning, anti-TH immunohistochemistry, and unbiased stereological TH-positive cell counting. All stereological counting was performed with the counter blinded to each mouse treatment.

### 4.7. Immunofluorescence

Cells fixed with 4% paraformaldehyde in PBS, fixed mouse brain samples, or postmortem human temporal lobe sections were blocked with a solution containing 5% normal donkey serum (Jackson Immuno Research Laboratories, West Grove, PA, USA), 2% BSA (Sigma-Aldrich, St. Louis, MO, USA), and 0.1% Triton X-100 (Sigma-Aldrich) for 1 h at room temperature. Samples were then incubated with combinations of primary antibodies against TH, GFAP, CD31, P-gp, α-synuclein, and pS129-αSyn—depending on the experiment—at 4 °C overnight. The fixed samples were then washed with PBS containing 0.1% Triton X-100 and incubated with corresponding secondary antibodies conjugated with fluorescent dye at room temperature for 1 h. Fluorescent images were obtained using a fluorescence microscope (Axiovert 200 M, Zeiss, Oberkochen, Germany).

### 4.8. Doxorubicin Uptake and Clearance Assay

HUVECs were plated in 12-well plates on glasses coated with poly-L-lysine (P1399, Sigma-Aldrich, St. Louis, MO, USA) at a density of 1 × 10^5^ cells per well. The cells were then preincubated with doxorubicin (10 μM, #44583, Sigma-Aldrich) for 2 h. The medium was removed and replaced with fresh medium with or without verapamil (50 μM, Sigma-Aldrich). After 3 h, the cells were washed with PBS and fixed with 4% paraformaldehyde for 15 min at room temperature. The cells were incubated with 4′,6-diamidino-2-phenylindole (DAPI) for 5 min at room temperature for nuclear counterstaining, and then observed using a fluorescence microscope (Axiovert 200 M, Zeiss). Randomly selected 3~4 cells from each captured image slide (6 image slides from 3 experiments) were used for doxorubicin fluorescence quantification. HUVECs viability in response to doxorubicin treatment was analyzed by Cell Counting Kit-8 (CCK-8) assay (#CK04-11, Dojindo Molecular Technologies, Rockville, MD, USA) according to the manufacturer’s instructions.

### 4.9. Acquisition and Handling of Human Postmortem Brains

Fixed postmortem human brain sections (temporal lobe) were provided by the Brain and Body Donation Program (BBDP) with approval of the material transfer agreement (17 May 2018). Fixed brain sections were subjected to immunofluorescence experiments as described above. The demographic information of the human samples is described in Table 1.

### 4.10. Statistical Analyses

Quantitative data are presented as mean ± standard error of the mean (SEM). Statistical significance was assessed using either the unpaired two-tailed Student’s *t*-test (two-group comparisons) or the analysis of variance (ANOVA) test with Tukey’s honestly significant difference post hoc analysis (more than three-group comparisons). Differences with a *p*-value of <0.05 were considered statistically significant. GraphPad Prism v. 5.03 (San Diego, CA, USA) was used for the preparation of all plots and statistical analyses.

## Figures and Tables

**Table 1:** Detailed information on postmortem fixed temporal lobe (TL) sections from human PD brains used in this study. Data for age and PMI were expressed as average ± standard deviation. Statistical differences were analyzed by the Chi-squared test for gender ratio, and by the unpaired two-tailed student’s *t* test for ages and postmortem interval between groups.

	Control	PD	*p* Value
N	6	6	
Male: female	3:3	3:3	0.56
Age (years)	80.83 ± 3.32	79.17 ± 2.06	0.68
Postmortem interval (days)	2.79 ± 0.49	3.47 ± 0.26	0.25

## Supplementary Materials

The following are available online at https://www.mdpi.com/1422-0067/21/22/8538/s1, Figure S1: Endothelial VDR downregulation in 6-OHDA PD mice are reversed by 1,25(OH)_2_D_3_ treatment; Figure S2: Endothelial VDR downregulation in PD mouse model of α-synucleinopathy; Figure S3: 1,25(OH)_2_D_3_ prevents PFF-induced downregulation of P-gp in HUVECs; Figure S4: Doxorubicin uptake and clearance in HUVECs.

## Abbreviations

MDR1	Multidrug resistance protein 1
VDR	vitamin D receptor
PD	Parkinson’s disease
P-gp	P-glycoprotein
PFF	α-synuclein preformed fibril
6-OHDA	6-hydroxydopamine
