# Supplementary material for: Brain Endothelial P-Glycoprotein Level Is Reduced in Parkinson’s Disease via a Vitamin D Receptor-Dependent Pathway
Source: Int J Mol Sci. 2020 Nov 12;21(22):8538. doi: 10.3390/ijms21228538 (PMC7696047; doi:10.3390/ijms21228538)
Supplement: Supplementary file 1 [file ijms-21-08538-s001.pdf]

SUPPLEMENTAL INFORMATION

Brain endothelial P-glycoprotein level is reduced in Parkinson's disease via a vitamin D receptor-dependent pathway

Hyojung Kim, Jeong-Yong Shin, Yun-Song Lee, Seung Pil Yun, Han-Joo Maeng, and Yunjong Lee

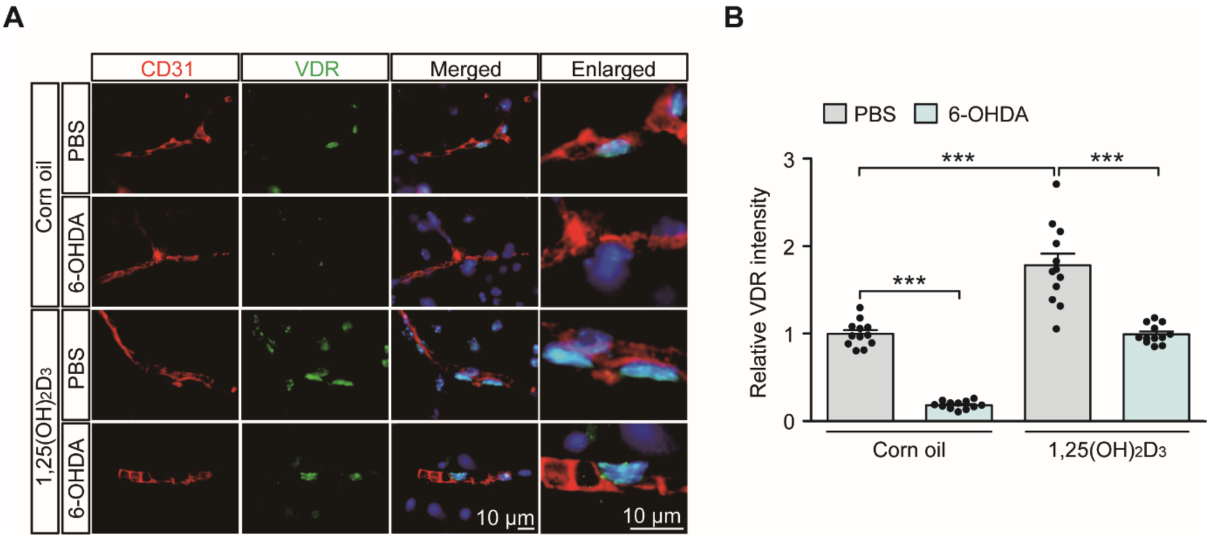

Supplementary figure 1. Endothelial VDR downregulation in 6-OHDA PD mice are reversed by 1,25(OH)$_2$D$_3$ treatment. (A) Representative immunofluorescence of VDR and CD31 in the ventral midbrain sections from each experimental mouse group. CD31 serves as a marker for endothelial cells. Scale bar = 10 μm. (B) Quantification of VDR immunofluorescence signal intensities in CD31-positive endothelial cells from the SNpc regions from each mouse group (*n* = 12 brain sections from 4 mice per group). Data are expressed as mean ± SEM. ***P < 0.001, ANOVA test, followed by Tukey's post hoc analysis.

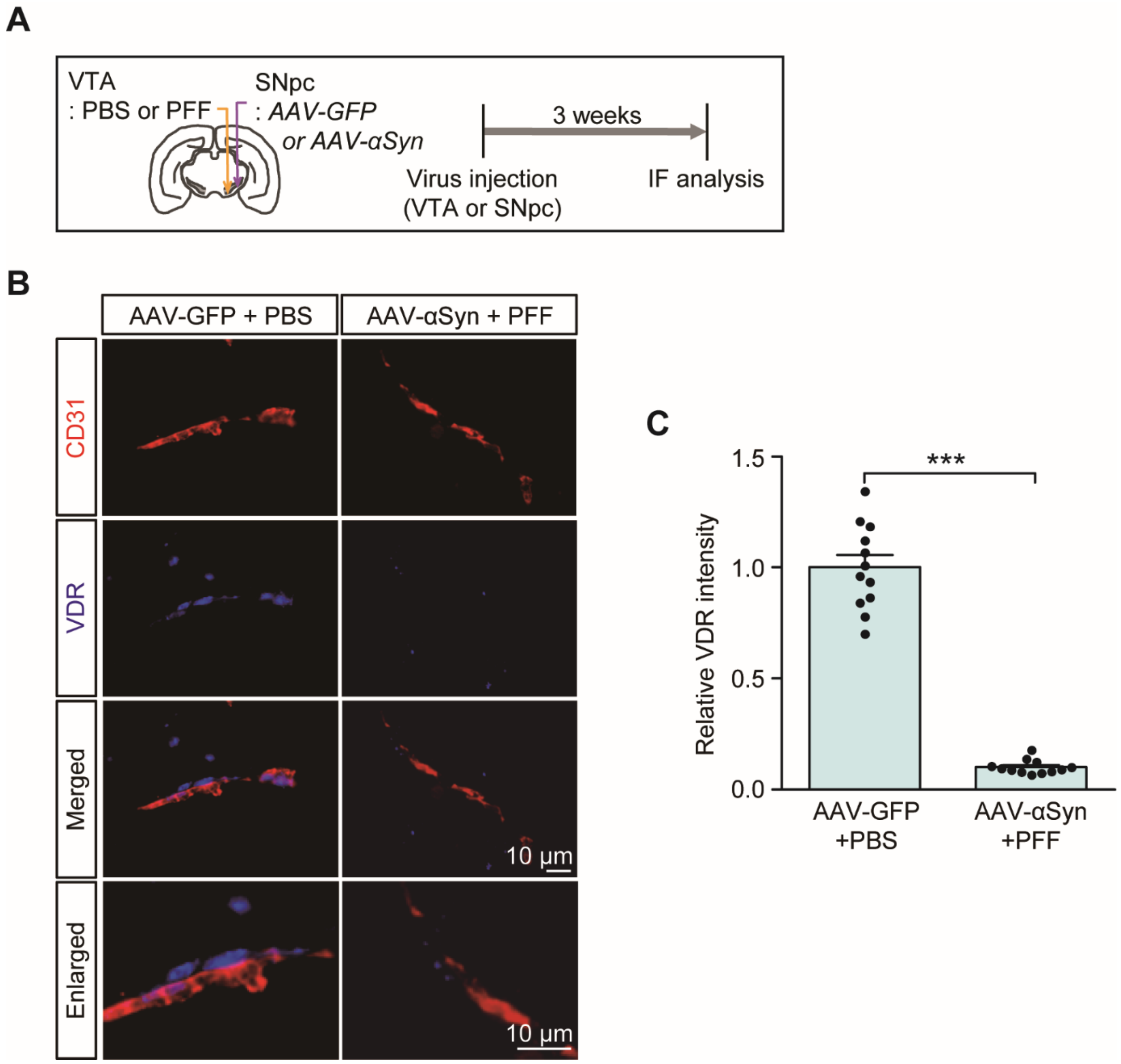

Supplementary figure 2. Endothelial VDR downregulation in PD mouse model of α-synucleinopathy. (A) Illustration of experimental schedule and combinatorial PFF/AAV-αSyn injections into VTA and SNpc of mouse brains. VTA, ventral tegmental area; SNpc, substantia nigra pars compacta; IF, immunofluorescence. (B) Representative immunofluorescence of VDR and CD31 in the ventral midbrain sections from each experimental mouse group. Scale bar = 10 μm. (C) Quantification of VDR immunofluorescence signal intensities in CD31-positive endothelial cells from the SNpc from each mouse group (n = 12 brain sections from 4 mice per group). Data are expressed as mean ± SEM. ***P < 0.001, unpaired two-tailed Student's t test.

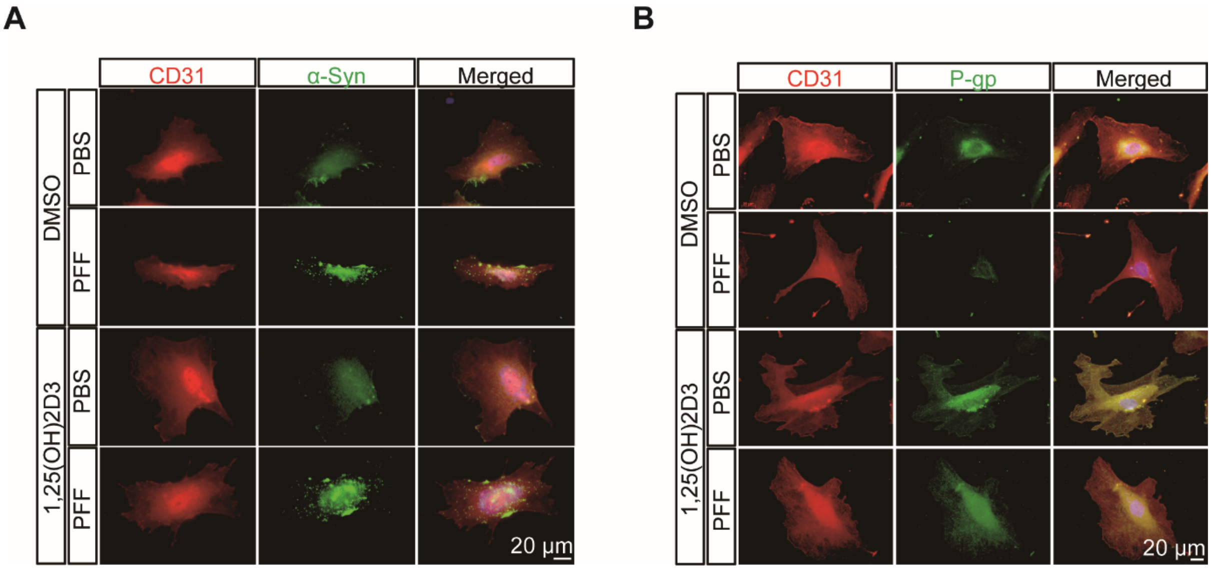

Supplementary figure 3. 1,25(OH)$_2$D$_3$ prevents PFF-induced downregulation of P-gp in HUVECs. (A) High magnification immunofluorescence images for experimental groups in the main Fig. 4C. Scale bar = 20 μm. (B) High magnification immunofluorescence images for experimental groups in the main Fig. 4E. Scale bar = 20 μm.

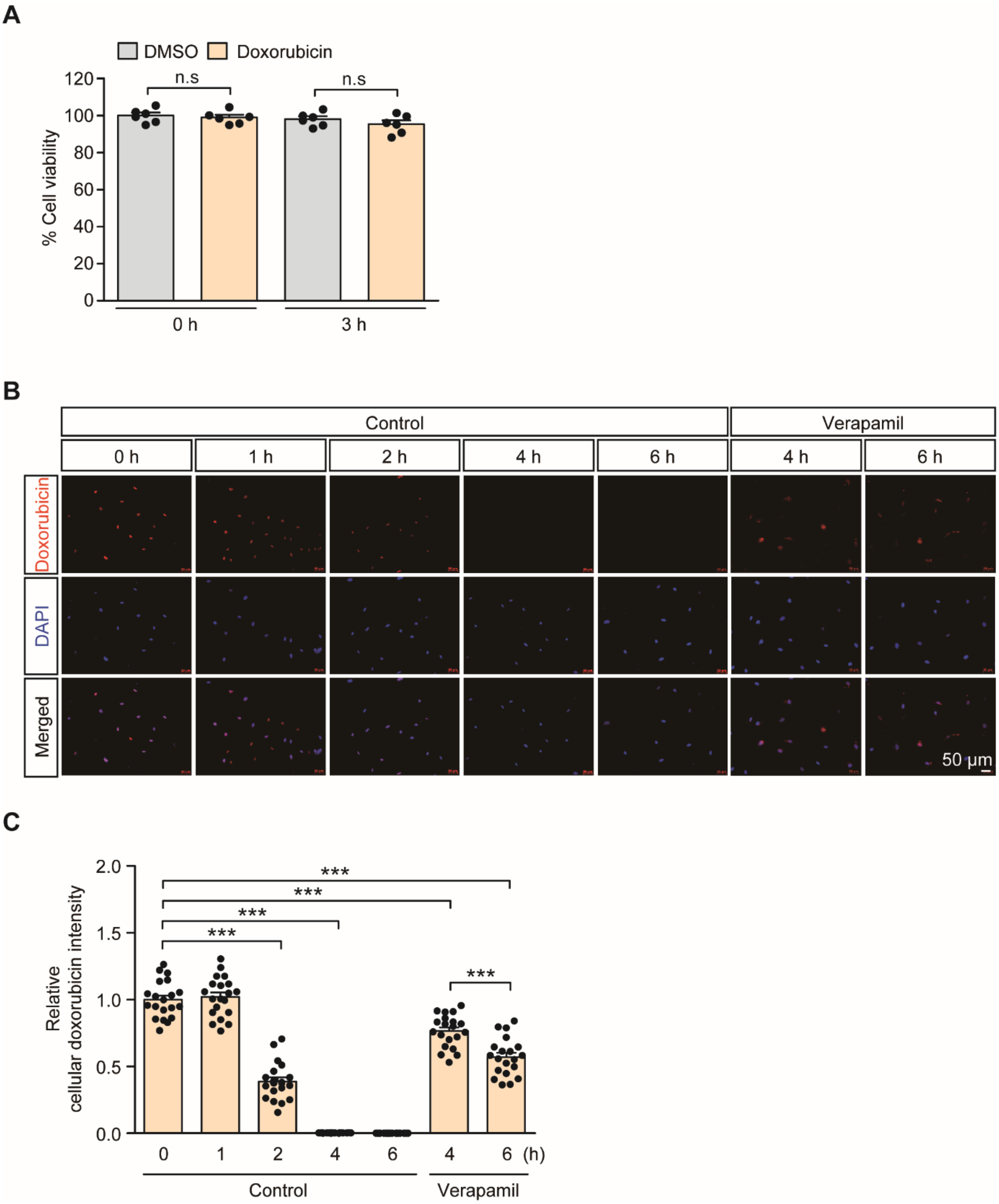

Supplementary figure 4. Doxorubicin uptake and clearance in HUVECs. (A) CCK-8 cell viability assessment in HUVECs (n = 6 per group). HUVECs were briefly treated with doxorubicin (10 μM, 2 h)) and maintained for the indicated time duration in the fresh media before the CCK-8 assay. (B) Representative fluorescence images of intracellular doxorubicin at the indicated time points (0, 1, 2, 4, and 6 h) in HUVECs following preincubation with 10 μM doxorubicin (2 h). Verapamil (50 μM) was added at 0 h to inhibit P-gp activity for the indicated duration. DAPI was used to counterstain the nucleus. (C) Quantification of relative intracellular doxorubicin autofluorescence in HUVECs at the indicated time points following 10 μM doxorubicin preincubation (2 h) (n = 20 cells from 3

experiments per group). Data are expressed as mean ± SEM. ***$P < 0.001$, ANOVA test, followed by Tukey's post hoc analysis. n.s., non-significant
